# Sodium taurocholate cotransporting polypeptide (NTCP) deficiency: Identification of a novel *SLC10A1* mutation in two unrelated infants presenting with neonatal indirect hyperbilirubinemia and remarkable hypercholanemia

**DOI:** 10.18632/oncotarget.22503

**Published:** 2017-11-18

**Authors:** Jian-Wu Qiu, Mei Deng, Ying Cheng, Raza-Muhammad Atif, Wei-Xia Lin, Li Guo, Hua Li, Yuan-Zong Song

**Affiliations:** ^1^ Department of Pediatrics, The First Affiliated Hospital of Jinan University, Guangzhou 510630, China

**Keywords:** sodium taurocholate cotransporting polypeptide, solute carrier family 10 member 1 gene (SLC10A1), hypercholanemia, hyperbilirubinemia, newborn

## Abstract

Sodium taurocholate cotransporting polypeptide (NTCP) is encoded by the gene *SLC10A1* and expressed in the basolateral membrane of the hepatocyte, functioning to uptake bile acids from plasma. Although *SLC10A1* has been cloned and NTCP function studied intensively for years, clinical description of NTCP deficiency remains rather limited. This study reported the genotypic and phenotypic features of two neonatal patients with NTCP deficiency. They both presented with neonatal indirect hyperbilirubinemia and remarkable hypercholanemia, and harbored the *SLC10A1* variants c.800C>T (p.S267F) and c.263T>C (p.I88T). On genetic analysis of the two family trios, the latter missense variant was detected in trans with the former, a reported loss-of-function variant. Having not been reported in any databases, the c.263T>C (p.I88T) variant demonstrated an allele frequency of 0.67% (1/150) in healthy controls. Moreover, this variant involved a relatively conservative amino acid, and was predicted to be pathogenic or deleterious by changing the conformation of the NTCP molecule. In conclusion, the novel variant c.263T>C (p.I88T) in this study enriched the *SLC10A1* mutation spectrum; the clinical findings lent support to the primary role of NTCP in hepatic bile acid clearance, and suggested that NTCP deficiency might be a contributing factor for the development of neonatal indirect hyperbilirubinemia.

## INTRODUCTION

In human beings, the liver uptake of bile salts is accomplished in sodium-independent and sodium-dependent manners. The former is mediated by the multiple organic anion transporting polypeptides (OATPs), including OATP1B1 and OATP1B3, while the latter, predominantly by the sodium taurocholate cotransporting polypeptide (NTCP) [1–3], which is encoded by the solute carrier (SLC) family 10 member 1 (*SLC10A1*) gene [4, 5]. As a transmembrane transporter in the basolateral membrane of hepatocytes, NTCP plays a vital role in the enterohepatic circulation of bile salts, being responsible for most of the hepatocellular sodium-dependent uptake of bile salts [6].

The human NTCP was cloned, localized and functionally characterized as early as in 1994 [7], and since then, some *SLC10A1* variants in human have been identified [8–11] while the NTCP function has also been studied intensively [8, 12]. Nevertheless, although NTCP deficiency has been predicted to result in hypercholanemia for years, previous reports of hypercholanemia-associated inherited disorders found causal mutations in genes other than *SLC10A1*, and these findings raised the possibility that an isolated NTCP gene defect may be asymptomatic, with limited impact on bile acid homeostasis [13]. In general, knowledge about the laboratory and clinical presentations of patients with NTCP deficiency remains rather limited in the past over 20 years. This situation, however, is changing in pace with the recent findings of patients with NTCP deficiency. As of today, 4 papers about NTCP deficiency have been published, involving a total of 6 patients comprising 3 children and 3 adult individuals. In 2015, Vaz *et al.* [14] reported the first child case of NTCP deficiency, who was homozygous for the *SLC10A1* mutation c.755G>A (p.R252H), with conjugated hypercholanemia being the prominent feature. In the year 2016, our team described the second child and the first adult with NTCP deficiency [15]. The 2-year-old patient had intractable and striking hypercholanemia, while the adult patient only had a slightly elevated serum bile acid level. Both patients were homozygous for c.800C>T (p.S267F), a loss-of function variant of *SLC10A1* gene [8]. Very recently, we diagnosed another infant and his mother [16] as 2 additional patients with NTCP deficiency, again both homozygous for the pathogenic variant c.800C>T (p.S267F). Although the mother only presented mildly increased plasma total bile acids (TBA), the infant exhibited persistent and remarkable hypercholanemia and cholestatic jaundice in early infancy. Moreover, Van Herpe *et al.* reported a 30-year-old female of Thai origin with NTCP deficiency as a compound heterozygote of the *SLC10A1* gene variants c.800C>T (p.S267F) and c.615-618del, also having persistently raised bile acids [17].

The above findings provided with important laboratory and clinical information for NTCP deficiency, with persistent hypercholanemia being the common presentation. However, as a new inborn error of metabolism of bile acids, NTCP deficiency is rather far from being completely understood, and the genotypic and phenotypic features yet remain open for investigation. As an example, the neonatal presentations of NTCP deficiency have never been reported thus far. This study described the clinical and molecular features of two unrelated neonates with NTCP deficiency. The two patients shared the same *SLC10A1* genotype with a novel mutation, and both had clinical and laboratory abnormalities since early neonatal period.

## RESULTS

### Clinical findings

*Patient 1.* A 9-day-old female infant was admitted to the Neonatal Section of our Department of Pediatrics due to jaundice over 6 days. At her age of 3 days, jaundiced skin and sclera was noticed, which was then aggravated progressively. As the second product of a non-consanguineous couple, the infant was spontaneously delivered at the gestation age of 38 weeks and 2 days, with a birth weight of 2.85 kg and a body length 48 cm. The Apgar score was 9 points at 1 min after umbilical ligation, and 10 points at 5 min, while the amniotic fluid was clear. The patient had a 9-year-old brother, who was physically healthy with normal social performance. The parents were healthy, and there was not family history of any genetic disease.

Physical examination found a body temperature (T) 36.5°C, heart rate (HR) 140 beats/min , respiratory rate (RR) 45 breaths/min, and a body weight (WT) 2940 g. Jaundice was observed in the skin and sclera. The anterior fontanelle was flat and soft, and no malformation of the head, ears, nose, mouth, and eyes could be observed. No positive signs were found in the two lungs and the heart. Her liver and spleen were not enlarged. The extremities were warm, and the distal perfusion was excellent.

After admission, the liver function test revealed indirect hyperbilirubinemia and markedly elevated serum TBA level. Her jaundice was alleviated soon in a response to phototherapy, but the TBA elevation persisted, even on the following-up in the clinic after she was discharged at the age of 19 days (Table 1). When aged 25 days, the infant underwent *SLC10A1* analysis to evaluate the possibility of NTCP deficiency. Sanger sequencing of the UGT1A1 gene was performed (Supplementary Table 1), but no pathogenic variant was detected.

**Table 1:** Biochemical indices in the two infants with NTCP deficiency and their parents

Biochemical indices^∆^	Patient 1						Parents		Patient 2						Parents	
	2D	9D	11D	18D	25D	69D	Father	Mother	5D	6D	8D	30D	33D	84D	Father	Mother
ALT (5–40 U/L)	–	27	–	28	32	68	49	21	8	11	12	15.3	13	41.7	58.7	12.6
AST (5–40 U/L)	–	46	–	–	55	100	35	34	22	26	21	23.5	20	48	34.9	16
GGT (8–50 U/L)	–	183	–	–	173	96	–	–	109	110	130	–	88	37.6	65.7	11.7
ALP (20–500 U/L)	–	279	–	–	330	432	–	–	169	159	–	–	277	312.5	49.9	102.7
TP (60.0–83.0 g/L)	–	54.6	–	–	53.2	54.6	76.2	–	54.1	55	–	–	49.9	60.5	73.8	76.2
ALB (35.0–55.0 g/L)	–	40	–	38	38.9	42.7	47.7	–	37.2	36.8	35.8	–	37.8	44.6	47.9	46.1
GLB (20.0–30.0 g/L)	–	14.6	–	–	14.3	11.9	28.5	-	16.9	18.2	–	–	12.1	15.9	25.9	30.1
Tbil (2–19 μmol/L^▲^)	195.9	346.5	244.1	103	72.5	53.3	–	–	310.1	301.2	99.2	40.8	29.4	6.6	10.7	12.3
Dbil (0–6 μmol/L)	9.6	22.4	14.3	12.2	13.4	14.4	–	–	10.3	21.3	11.8	7.5	9.7	1.2	2.0	1.7
Ibil (2.56–20.9 μmol/L)	186.3	324.1	229.8	90.8	59.1	38.9	–	–	299.8	279.9	87.4	33.3	19.7	5.4	8.7	10.6
TBA (0–10 μmol/L)	–	66.2	–	93.3	49.6	88.8	–	–	83.9	95.1	75.8	157.9	59.7	42.7	10.0	4.1
AFP (0–20 ng/mL^▼^)	–	–	–	–	4096.3	1012	–	–	–	–	–	–	–	115.8	–	–
Ferritin (10–291 ng/mL)	–	–	–	–	899.5	458	–	–	–	–	–	–	–	74.7	–	–
25OHD (30–100 ng/ml)	–	–	–	–	16.9	16.3	–	–	–	–	–	–	–	44.1	–	–

D, days ;ALT, alanine transaminase; AST, aspartate transaminase; GGT, gamma-glutamyl transpeptidase; ALP, alkaline phosphatase; TP, total protein; ALB, albumin; GLB, globulin; Tbil, total bilirubin; Dbil, direct bilirubin; Ibil, indirect bilirubin; TBA, total bile acids; AFP, alpha-feto protein; 25-OH-VitD3,25-hydroxyvitamin D3; –, not tested.

^∆^Within brackets behind the relevant indices were their reference ranges.^▲^The Tbil reference range after 3 weeks of life. In full-term baby, the Tbil upper limit in the first 1–4 days were 85, 145, 190, and 215 μmol/L, respectively; and in the 5–7 days of life, 225 μmol/L. Tbil should subside to normal range within 3 weeks of life.^▼^The AFP reference range for childhood and adulthood period.

*Patient 2.* A 5-day-old male infant was admitted to our Neonatal Section because of jaundice for 3 days. Mild jaundice appeared at age 2 days. At the 5th day after birth, the total bilirubin increased to 310.1 μmol/L. Then the infant was referred to our clinic, where a laboratory test revealed elevated TBA as well as indirect hyperbilirubinemia (Table 1), and thus the infant was admitted for further management. As the first child of a non-consanguineous couple, the patient was delivered vaginally at the gestational age of 37 weeks with the birth weight 3050 g. The parents were both healthy. Family history of any genetic disease was denied.

On physical examination, the body weight was 2910 g, with T 36.2°C, HR 130 beats/min, and RR 43 breaths/min. Jaundiced skin and sclera were observed. The lungs were clear on auscultation, and no abnormal heart sound or murmur was heard. There was no abdominal distention, and the liver and spleen were not palpable. Primitive reflexes were normal and no pathological reflexes could be found on nervous system examination.

The patient underwent phototherapy for 3 days. As a result, his jaundice subsided, and he was discharged at age 9 days. No special treatment was given thereafter, and the jaundice did not reappear. However, his serum TBA levels kept high in the following 1 month, and hence *SLC10A1* analysis was performed at age 33 days to evaluate the possibility of NTCP deficiency. On Sanger sequencing of the UGT1A1 gene, no pathogenic variant was detected but three benign SNPs in the 3ʹ UTR, i.e. rs10929303, rs1042640 and rs8330 [https://www.ncbi.nlm.nih.gov/SNP/].

### *SLC10A1* genotypes

**Figure 1:**
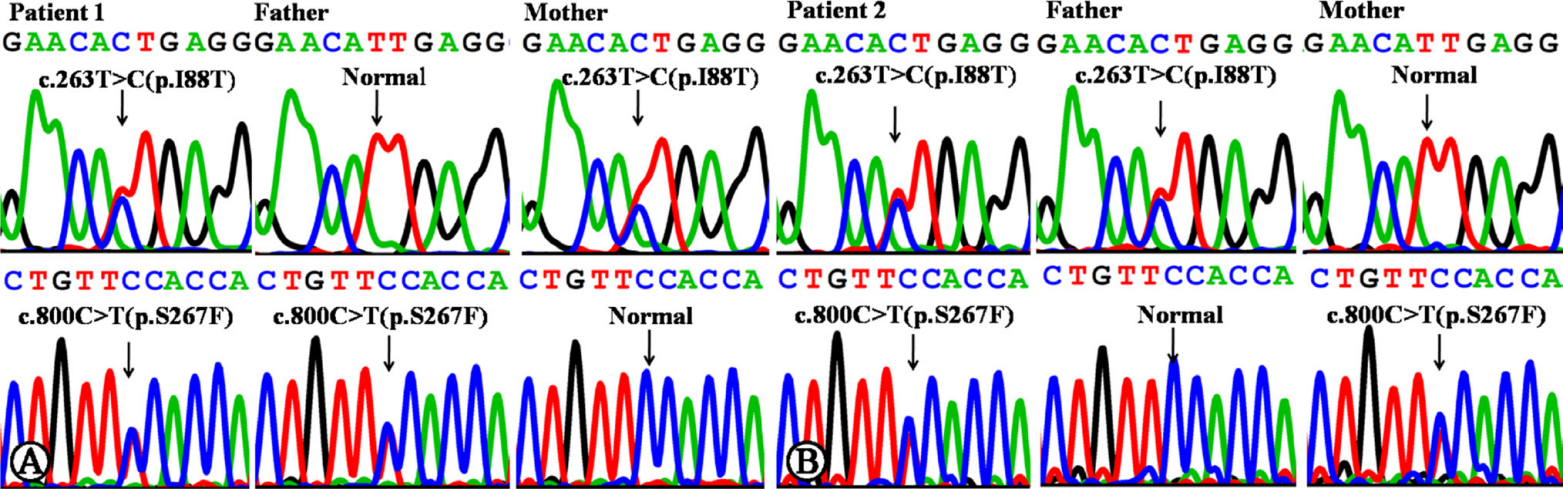
*SLC10A1* gene analysis in the two families affected by NTCP deficiency (**A**) Patient 1 was a compound heterozygote of mutation c.800C>T (p.S267F) and c.263T>C (p.I88T). Her father was a carrier of c.800C>T, while the mother, of c.263T>C. (**B**) Patient 2 was a compound heterozygote of mutation c.800C>T (p.S267F) and c.263T>C (p.I88T), too. His father was a carrier of c.263T>C, while the mother, of c.800C>T.

Sanger sequencing of the *SLC10A1* gene demonstrated that both infants were compound heterozygotes of the variants c.800C>T (p.S267F) and c.263T>C (p.I88T). The father of patient 1 was a carrier of variant 800C>T (p.S267F), and the mother, a carrier of c.263T>C (p.I88T) (Figure 1A). The father and mother of patient 2 were carriers of c.263T>C (p.I88T) and c.800C>T (p.S267F), respectively (Figure 1B). To the best of our knowledge, the variant c.263T>C (p.I88T) has not been reported in any other references in the Pubmed database, and has not as yet been included in 1000 Genomes Project, Exome Sequencing Project, Exome Aggregation Consortium, and Human Gene Mutation Database.

### Polymerase chain reaction (PCR)-restriction fragment length polymorphism (RFLP)

**Figure 2:**
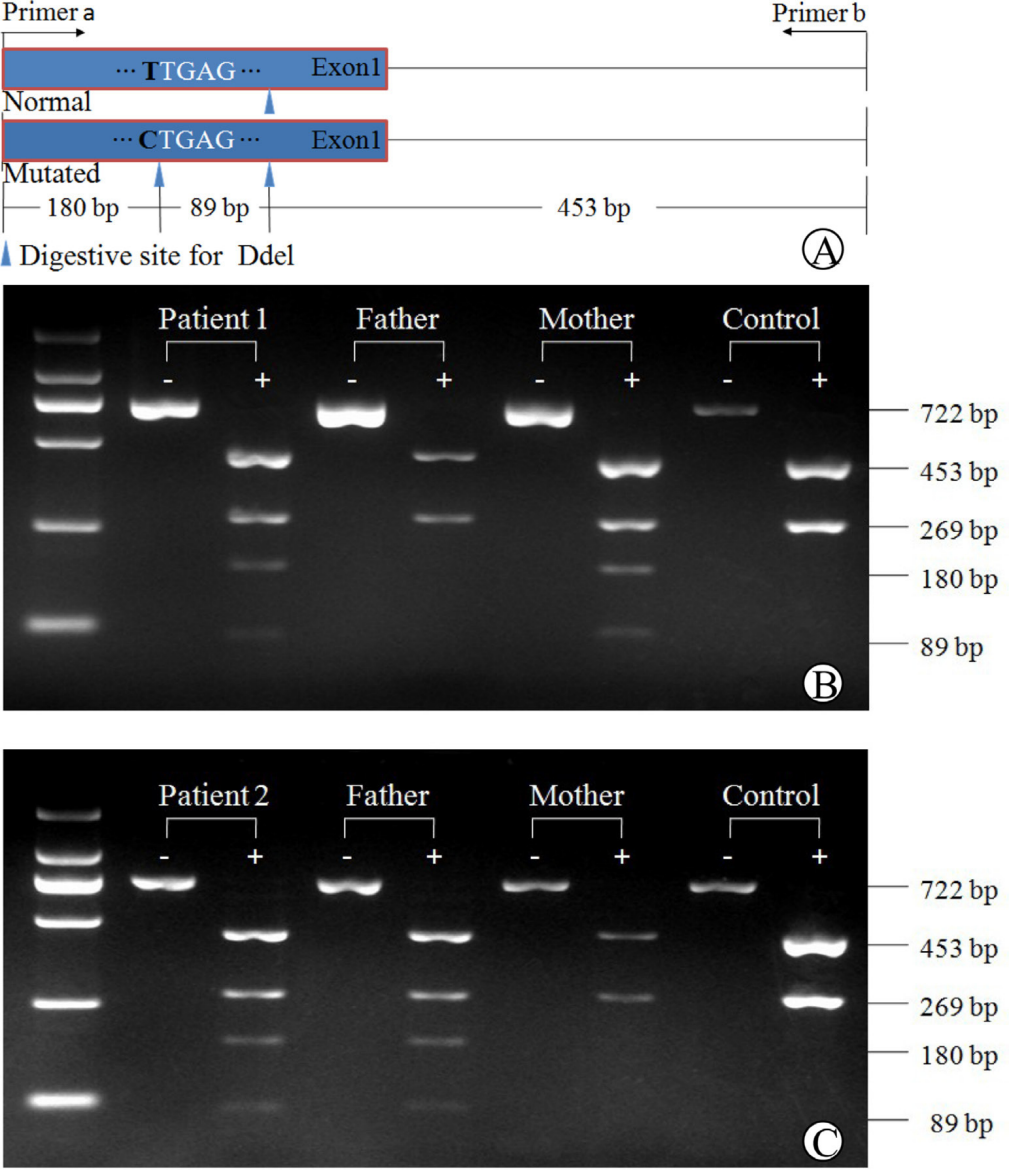
PCR-RFLP analysis of the *SLC10A1* variant c.263T>C (p.I88T) in the two families The schematic diagram of the PCR-RFLP procedure was illustrated in (**A**). Electrophoresis of the PCR-RFLP products (**B**) demonstrated that patient 1 and her mother were both carriers of c.263T>C, harboring two additional bands of 89 bp and 180 bp arising from Ddel digestion of the mutated PCR products. Her father had the same DNA bands of 453bp and 269bp as in the healthy control. Similarly in (**C**), patient 2 and his father were both carriers of c.263T>C, while the mother was negative for this variant.

A PCR-RFLP procedure, as illustrated in Figure 2, was developed to confirm the *SLC10A1* genotypes in the two families, and to investigate the allele frequency of the new variant c.263T>C (p.I88T). The PCR-FRLP findings, as shown in Figures 2B and 2C, were consistent with that of Sanger sequencing — both infants were heterozygous for the variant c.263T>C (p.I88T), which was inherited from the mother in family 1, and from the father, in family 2. In addition, only one carrier was detected on the screening for the variant in the 75 control subjects, and thus the carrier rate was calculated to be 0.67% (1/150), indicating that this missense variant is a novel *SLC10A1* mutation.

### Bioinformatic analyses

**Figure 3:**
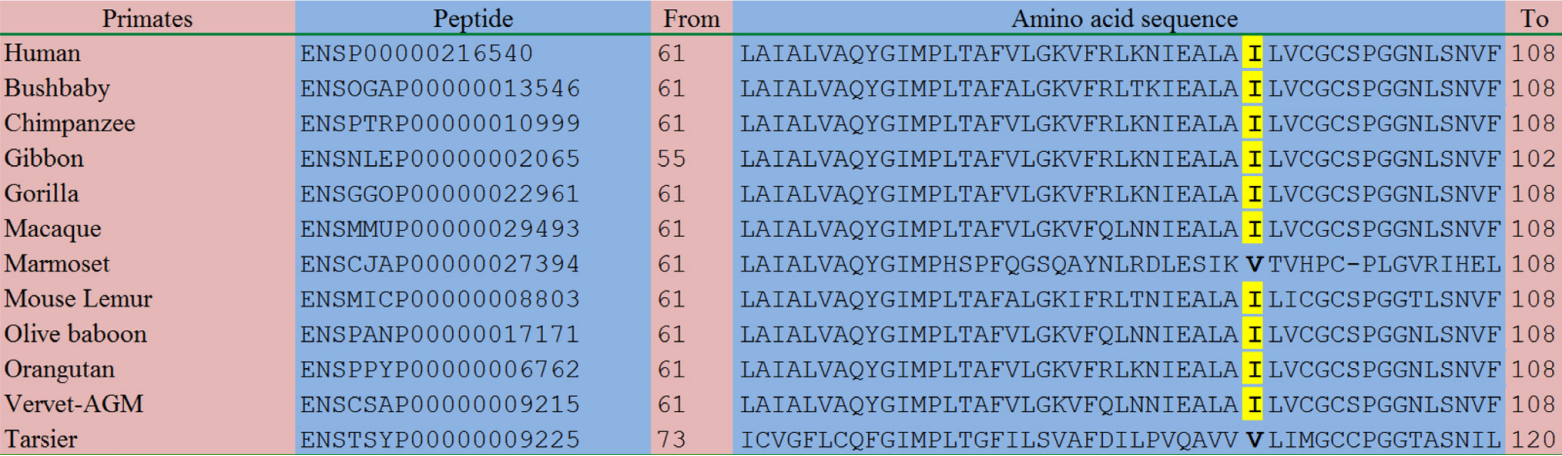
Comparative alignment of the homologous peptides in diverse species With the exception of Marmoset and Tarsier, all the remaining 10 species of primates, including Human, Bushbaby, Chimpanzee, Gibbon, Gorilla, Macaque, Mouse Lemur, Olive baboon, Orangutan, and Vervet-AGM, had the same isoleucine residue as highlighted in yellow. This finding indicated that the novel c.263T>C (p.I88T) variant affected a relatively conserved amino acid residue.

As shown in Figure 3 as well as Supplementary Figure 1, the amino acid sequences of the homologous peptides in a total of 83 species were aligned comparatively. Although the amino acid p.88Ile was not so conserved in other vertebrates and other species (Supplementary Figure 1), this residue was relatively conserved in primates and mammals: in 9/12 of the primates including human (Figure 3), 19/22 of the rodents and lagomorphs, and 22/24 of the other mammals (Supplementary Figure 1), this residue kept consistent.

On ployphen-2 analysis, the variant c.263T>C (p.I88T) was predicted to be “possibly damaging” with a score of 0.772. When predicted using PROVEAN, the score was -3.165, also indicating a “deleterious” variant. Moreover, this variant was predicted to be “deleterious” on SIFT prediction with a score of 0.007.

**Figure 4:**
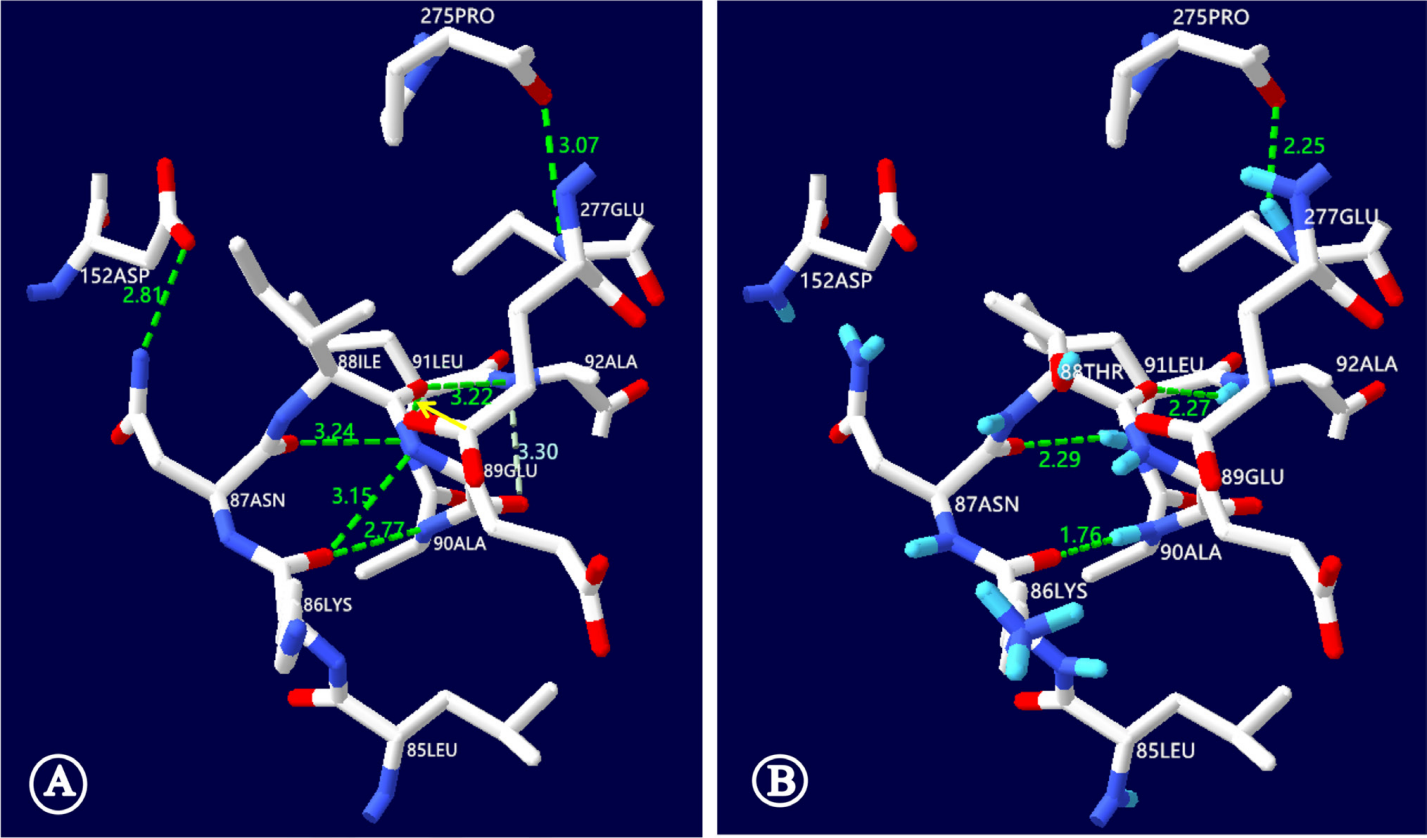
NTCP molecular alteration caused by the *SLC10A1* variant c.263T>C (p.I88T) This figure illustrated the ball-and-stick model of human NTCP, with the short bar in white, red and blue standing for the carbon, oxygen and nitrogen atoms, while the dashed lines in green illustrated the hydrogen bonds, respectively. (**A**) In the wild-type model, the NTCP conformation was maintained by a series of hydrogen bonds, including the one between the isoleucine at position 88 and the leucine at 91 (denoted by the yellow arrow). (**B**) In the mutant NTCP model, the isoleucine at position 88 was changed to a threonine (p.I88T). This change led to disappearing of the hydrogen bond between the positions 88 and 91, and shortened the distance of the hydrogen bond between the positions 88 and 92 from 3.22 to 2.27 nm. In addition, the NTCP conformation was further distorted by other hydrogen bond alterations. Among them, the hydrogen bond between the leucine at 91 and the asparagine at 87 was shortened from 3.24 to 2.29nm while the hydrogen bond disappeared between the lysine at 87 and the glutamic acid at 89. ILE: isoleucine; THR: threonine; ALA: alanine; LEU: leucine; ASN: asparagines; LYS: lysine; GLU: glutamic acid; ASP: aspartic acid; PRO: proline.

The c.263T>C (p.I88T) mutation resulted in the replacement of a hydrophobic isoleucine by a hydrophilic threonine at the amino acid position 88 of the NTCP molecule. This replacement, as illustrated in Figure 4, led to NTCP conformation alteration and hence might affect the function of NTCP to uptake bile acids. On NTCP protein structural model analysis, the hydrogen bond disappeared between the isoleucine at position 88 and the leucine at 91, while the distance of the hydrogen bond between the isoleucine at position 88 and the alanine at 92 was shortened from 3.22 nm to 2.27 nm. Moreover, the NTCP conformation could be further distorted by additional hydrogen bond changes among other amino acid residues of this carrier protein (Figure 4).

## DISCUSSION

In this study, two unrelated infants were diagnosed to have NTCP deficiency by way of Sanger sequencing of the *SLC10A1* gene. The patients were both compound heterozygotes of the missense variants c.800C>T (p.S267F) and c.263T>C (p.I88T), and their parents were all carriers of the relevant variants. In the two families, the c.263T>C (p.I88T) variant was detected in trans with c.800C>T (p.S267F), a *SLC10A1* variant that had proven pathogenic by functional, bioinformatic, and clinical evidences [10, 15–17]. The c.263T>C variant has not been included in 1000 Genomes Project, Exome Sequencing Project, Exome Aggregation Consortium, and Human Gene Mutation Database, and its allele frequency was calculated to be 0.67% in this study. In addition, bioinformatic analysis using a variety of *in silico* tools, including function prediction, homologous protein alignment and NTCP protein structure analysis, suggested that the variant c.263T>C involves a conserved amino acid, and might be deleterious/damaging by changing the conformation of the NTCP molecule. Another possibility could not be ruled out that this variant might give rise to NTCP transport defect to the cell surface, a targeting problem as in the cases of other *SLC10A1* variants [8,14]. Moreover, although the available data for the liver function indices were incomplete for the parents of patient 1, this novel missense mutation demonstrated cosegregation with hypercholanemia in family 2 (Table 1). Although functional analysis of the *SLC10A1* variant c.263T>C were not carried out in this study due to technical limitation, the above findings together strongly supported the diagnosis of NTCP deficiency in our patients. So far as we know, the two infants in this study were the youngest patients with NTCP deficiency as of today.

Similar to previously reported cases of NTCP deficiency, both patients in this paper exhibited persistent hypercholanemia. Of note, besides the persistently raised serum TBA levels, they both experienced neonatal indirect hyperbilirubinemia, as shown in Table 1. This finding suggested that NTCP deficiency might work as one of the contributing factors that affect the bilirubin homeostasis, giving rise to the development of indirect hyperbilirubinemia, particularly in neonates who have very rich bilirubin sources but immature liver function to uptake, conjugate and excrete bilirubin [18, 19]. Actually, the organic anion transport polypeptides (OATPs), which belong to the solute carrier organic anion transporter (*SLCO*) superfamily [20], are responsible for the uptake of plasma bilirubin (direct and indirect) across the basolateral membrane of the hepatocyte, and this transporter also has the function to uptake bile acids into the hepatocyte [21]. Therefore, the elevated plasma bile acids in NTCP-deficient patients will competitively inhibit the OATPs function to uptake direct and indirect bilirubin, leading to neonatal indirect hyperbilirubinemia as in the patients in this study, or causing cholestatic jaundice in infants as reported previously [16]. It was noteworthy that NTCP deficiency might not be the unique causative factor for indirect hyperbilirubinemia in the two infants, since the total bile acid levels kept beyond the upper limit of the reference range while hyperbilirubinemia got alleviated or resolved gradually in both patients (Table 1).

Moreover, our patients in this paper showed other laboratory changes. The serum levels of alpha-fetoprotein (AFP) were dramatically elevated in both patients. It was well known that serum AFP levels in newborns at birth was very high, with a reference interval of 15700–146,500 ng/ml and a medium value of 48,300 ng/ml [22], which then decreased dramatically with the increased age, and reached the childhood or adulthood reference range by around 6 weeks [23] to 8 months [24], and even by 3 years of age [25]. The age-dependant wide variation range of AFP levels indicated that there must be more than one unknown factor affecting the AFP serum level [24]. Moreover, patient 1 had at day 69 elevated transaminases, especially AST, which are about two-fold above the normal value, and the ferritin level in the same infant was also dramatically increased. Taking these changes together, such a possibility could not be completely ruled out that NTCP deficiency might be one of the factors disturbing the hepatocellular function via unknown mechanisms in the two infants. Moreover, the serum level of 25-OH-VitD_3_ was decreased in patient 1. Overall, the relationship between these changes and NTCP deficiency remains an enigma, and the diagnosis of more such patients might facilitate the resolving of this issue.

The diagnosis of the two patients in this study further support the primary role of NTCP in hepatic bile acid clearance, but raised several unanswered questions at the same time. First, it remains elusive whether hypercholanemia caused by NTCP deficiency needs to be actively addressed. To date, no severe clinical implication was found in neonates with NTCP deficiency, and the intestinal bile acid signaling remained normal, as evidenced by plasma FGF19 levels [15]. However, since this disease caused remarkable and persistent hypercholanemia while bile salt synthesis in the liver was not affected [15], it might not be unreasonable to consider bile acid accumulation in enterocytes and in the luminal ileum, which had been reported to increase the risk of necrotizing enterocholitis [26]. And, interference with NTCP expression could reduce gallbladder bile volume and increase the susceptibility to cholesterol gallstones [27]. Second, NTCP deficiency might be an issue of significance in terms of personalized medicine. NTCP transports a variety of substrates other than bile salts, including steroid and thyroid hormones as well as some xenobiotics such as statins, diagnostic contrast agent and some antifungal agents [28, 29]. Third, it also remains unclear whether or not NTCP deficiency will benefit the affected individuals in terms of HBV infection. NTCP has been found to be the cell surface receptor necessary for the hepatocytic entry of HBV/HDV [30, 31]. Although the *SLC10A1* variant p.S267F is independently associated with the decreased risk of cirrhosis and HCC as well as resistance to CHB infection [32], there was another report showing no associations of *SLC10A1* variants with susceptibility to persistent HBV infection among Southern Chinese [33], and some genetic variant of NTCP may be associated with increased risk of HBV infection [11]. Careful follow-up of patients with NTCP deficiency might provide insights relevant to these unresolved issues.

In summary, by way of clinical and *SLC10A1* genetic analysis, we diagnosed two unrelated infants with NTCP deficiency, who presented with indirect hyperbilirubinemia and marked hypercholanemia as early as in the neonatal period. The novel missense variant c.263T>C (p.I88T) enriched the *SLC10A1* mutation spectrum. The clinical findings in this study lent support to the primary role of NTCP in hepatic bile acid clearance, and suggested that NTCP deficiency might be a contributing factor for the development of neonatal indirect hyperbilirubinemia.

## MATERIALS AND METHODS

### Subjects and ethics

The research subjects in this study were two infants with elevated serum bilirubin and TBA as well as their parents. The first infant was a female aged 9 days, and the second a male at 5 days of life. Their clinical data, including the chief complaints, history, symptoms, signs, and laboratory results, were collected from the record data system in our hospital. The clinical findings were described as case reports. In order to explore the allele frequency of the identified novel *SLC10A1* mutation, 75 blood samples (with a total of 150 *SLC10A1* alleles) from health volunteers were collected as the controls.

This research was carried out with written informed consents from the parents of the patients and all the volunteers. The study has been approved by the Committee for Medical Ethics, the First Affiliated Hospital, Jinan University in Guangzhou China, and adhering to the World Medical Association Declaration of Helsinki (WMADH 2008).

### Sanger sequencing

Genomic DNA was extracted by using a DNA extraction kit (Simgen, China,) according to the manufacturer’s instructions. And then, Sanger sequencing of all the 5 *SLC10A1* exons and their flanking sequences were carried out, using the DNA fragments amplified by PCR. The primers and polymerases used for PCR amplification were listed in Table 2.

**Table 2:** Primers for amplification and sequencing analysis and the PCR conditions

Targets	NTCP primer sequences(5′ to 3′)	AT (°C)	Polymerase	Products (bp)
Promotor^▲^	Forward:CACAGTAGGAGGTGGAAGGATTTTG	58	LA-Taq	2117
	Reverse: CTTGCTGGATGCCTTCTTTAATC			
Exon 1	Forward: GAAACTAAGGAATCAAGAGCGGAGC	58	Taq	1248
	Reverse: CAGGAATTTGAGGTGCTCATTTGG			
Exon 2	Forward: CCACTTACTACCTTGTGCGACTTTG	58	Taq	991
	Reverse: TGGAATTGGATCTTGTTTCTCTCG			
Exon 3	Forward: CACACCTGTAATCCCAGCACTTTGG	58	Taq	983
	Reverse: GTGTTTGGATACCTTTGGTGTCTG			
Exon 4	Forward: CACTTTCCTGGCAATATGTTCAGATG	58	Taq	628
	Reverse: GATGGAAGTAGTCTTGGATCTTTAATG			
Exon 5	Forward: CGAAGTTAGAAGTGAAGTGATGATGAAG	58	Taq	1432
	Reverse: CTGTGTTTCTCGTTTTGGTGTTGG			

AT, annealing temperature; bp, base pairs.^▲^For the sequencing of the promotor region, an additional primer was used with the nucleotide sequence 5ʹ-CCTAGGATAACCTCACACACTAG-3ʹ.

All PCR amplification was performed in a mixture of 50 μL of total volume, containing 5 μL of 10× Buffer (Mg^2+^ plus) (TaKaRa, China), 4 μL of dNTP Mixture (2.5 mM), 37.75 μL of sterilized distilled water (PCR-grade), 0.25 μL of Taq (5 U/µl, TaKaRa, China), 2 μL of the forward and reverse primers (10 mM) together, and 1 μL of genomic DNA template. The products were then analyzed by Sanger sequencing on a 96-capillary ABI 3730xl DNA Analyzer (Applied Biosystems, Thermo Fisher Scientific, USA) with a BigDye Terminator v 3.1 Cycle Sequencing Kit (Thermo Fisher Scientific, USA), according to the manufacturer’s instructions. The sequencing results were aligned with the *SLC10A1* cDNA sequence, which was available at http://www.ensembl.org/ by using a DNAman software version 5.2.2 (Lynnon BioSoft Corporation, USA). The allele frequency of the identified novel *SLC10A1* variant was checked among such population databases as 1000 Genomes Project (http://browser.1000genomes.org), Exome Sequencing Project (https://esp.gs.washington.edu/drupal/), Exome Aggregation Consortium (http://exac.broadinstitute.org/), and The Human Gene Mutation Database (http://www.hgmd.cf.ac.uk/ac/index.php).

### PCR-RFLP approach

A new PCR-RFLP procedure was developed to confirm the *SLC10A1* genotypes of all family members and to screen for the novel *SLC10A1* variant c.263T>C (p.I88T) in 75 healthy individuals. The sequences of the forward and backward primers for PCR amplification of DNA fragment with the novel variant were 5′-cgtcatcctggtgttcatgttgttc-3′ and 5′-caggaatttgaggtgctcatttgg-3′, respectively (Invitrogen, China). The PCR temperature profile was 94°C for 5 min, followed by 35 cycles of 94°C for 40 sec, 62°C for 50 sec and 72°C for 30 sec, and a final extension step 72°C for 10 min. The restriction enzyme in RFLP analysis was Ddel (Promega Corporation, USA). For frequency calculation of the novel variant, the number of mutated alleles detected in all 75 control samples was divided by 150, and then the quotient was amplified by 100%.

### Alignment of homologous peptides

Amino acid sequences of 83 homologous peptides for NTCP were collected by the orthologue list of the human *SLC10A1* gene in the Ensembl Genome Browser (http://www.ensembl.org). The 83 species were then classified into five taxonomy subgroups: primates, rodents and lagomorphs, other mammals, other vertebrates, and other species, and aligned using BLAST/BLAT Ensembl software (http://www.ensembl.org/Multi/Tools/Blast?db=core). The 12 species of primates in this paper encompassed Human, Bushbaby, Chimpanzee, Gibbon, Gorilla, Macaque, Marmoset, Mouse Lemur, Olive baboon, Orangutan, Vervet-AGM, and Tarsier; The 22 rodents and lagomorphs included Mouse, Ryukyu mouse, Shrew mouse, Pika, Rabbit, Brazilian guinea pig, Chinese hamster CriGri, Chinese hamster CHOK1GS, Damara mole rat, Degu, Golden hamster, Guinea pig, Kangaroo rat, Long-tailed Chinchilla, Naked mole-rat male, Naked mole-rat female, Northern American deer mouse, Prairei love, Rat, Lesser Egyptian jerboa, Squirrel, and Upper Galilee mountains blind mole rat; The 24 other mammals were Cat, Dog, Ferret, Panda, Alpaca, Cow, Dolphin, Horse, Pig, Sheep, Armadillo, Elephant, Hedgehog, Hyrax, Lesser hedgehog tenrec, Megabat, Microbat, Shrew, Sloth, Tree Shrew, Opossum, Platypus, Tasmanian devil, and Wallaby; The 21 other vertebrates included Anole lizard, Chinese softshell turtle, Chicken, Duck, Flycatcher, Turkey, Zebra Finch, Amazon molly, Cave fish, Cod, Coelacanth, Fugu, Medaka, Platyfish, Spotted gar, Stickleback, Tetraodon, Tilapia, Zebrafish, Xenopus, and Lamprey; The 4 other species consisted of Caenorhabditis elegans, Ciona intestinalis, Ciona Savignyi, and Fruitfly.

### *In silico* prediction of pathogenicity

Three prediction programs were used to predict the pathogenicity of the novel *SLC10A1* variant c.263T>C (p.I88T) identified in the two patients. PROVEAN (Protein Variation Effect Analyzer) (http://provean.jcvi.org/seq_submit.php) predicts for variant as “probably damaging” if the probability is <–2.5 [34] PolyPhen-2 (http://genetics.bwh.harvard.edu/pph2/) analysis identifies variant as “probably damaging” if the probability is >0.85, and as “possibly damaging”, with the probability >0.15 [35]. SIFT (http://sift.jcvi.org/www/SIFT_chr_coords_submit.html) classifies the variant as being “deleterious” provided that the SIFT score is < 0.05 [36].

### Structural effect of the novel mutation on NTCP protein

To evaluate the structural change of the NTCP protein caused by the identified novel *SLC10A1* mutation c.263T>C(p.I88T), the affected NTCP molecule was constructed using the SWISS-MODEL automated protein modeling server (https://swissmodel.expasy.org/), as described previously [37–39], and the PDB structure 4n7w.2.A (https://swissmodel.expasy.org/templates/4n7w.2), which describes the crystal structure of the sodium bile acid symporter from Yersinia frederiksenii, was herein used as a template for the NTCP molecule with the amino acid change due to the identified novel *SLC10A1* mutation. The NTCP molecular structure was viewed using the software SWISS-Pdb Viewer 4.1(http://spdbv.vital-it.ch/index.html), and the structural changes raised by the *SLC10A1* variant was observed in the lowest energy conformation.

## SUPPLEMENTARY MATERIALS FIGURE AND TABLE


