# Supplementary material for: Sodium taurocholate cotransporting polypeptide (NTCP) deficiency: Identification of a novel SLC10A1 mutation in two unrelated infants presenting with neonatal indirect hyperbilirubinemia and remarkable hypercholanemia
Source: Oncotarget. 2017 Nov 18;8(63):106598–607. doi: 10.18632/oncotarget.22503 (PMC5739759; doi:10.18632/oncotarget.22503)
Supplement: Supplementary file 1 [file oncotarget-08-106598-s001.pdf]

Sodium taurocholate cotransporting polypeptide (NTCP) deficiency: Identification of a novel *SLC10A1* mutation in two unrelated infants presenting with neonatal indirect hyperbilirubinemia and remarkable hypercholanemia

SUPPLEMENTARY MATERIALS

Supplementary Table 1: PCR primers and conditions for the sequencing analysis of UGT1A1 gene*

Targets	Primer sequences (5′ to 3′)	AT (˚C)	Polymerase	Products (bp)
Promotor	Forward: AATGGATCCTGAGGTTCTGG	58	Taq	834
	Reverse: CTGGGTAGCCTCAAATTCCA			
Exon 1	Forward: AGGAGCAAAGGCGCCATGGC	58	Taq	1042
	Reverse: TGCCAAAGACAGACTCAAACC			
Exon 2	Forward: AACACGCATGCCTTTAATCATA	56	Taq	416
	Reverse: TGACAACAACCACAACAACAAA			
Exon 3	Forward: GAAGTTGCCAGTCCTCAGAAG	56	Taq	356
	Reverse: TGTTGGCCATAATATTTTCAAGC			
Exon 4	Forward: AACACTGAGTCTTTGGAGTGTTTTC	56	Taq	420
	Reverse: TATTTGAAACAACGCTATTAAATGCT			
Exon 5	Forward: CAGGTTTCCTTTCCCAAGTTT	60	Taq	1280
	Reverse: GAAGGCGTGTGTGTGTGAAC			

AT, annealing temperature; bp, base pairs.

*Based on the reference: Minucci A, Canu G, Gentile L, *et al*. Identification of a novel mutation in UDP- glucuronosyl transferase (UGT1A1) gene in a child with neonatal unconjugated hyperbilirubinemia. Clinical Biochemistry. 2013; 46:170–172.

Rodents and lagomorphs	Peptide	From	Amino acid sequence			To
Mouse	ENSMUSP00000093229	61	VIIAIVAQYGIMPLSAFLLGKVFHLTSIEALA	I	LICGCSPGGNLSNLF	108
Ryukyu mouse	MGP_CAROLIEiJ_P0032680	61	VIIAIVAQYGIMPLSAFLLGKVFHLTSIEALA	I	LICGCSPGGNLSNLF	108
Shrew mouse	MGP_PahariEiJ_P0082793	61	VIIAMVAQYGIMPLSAFLLGKVFRLTNIEALA	I	LICGCSPGGNLSNLF	108
Pika	ENSOPRP00000007592	61	LAIALVAQYGIMPLTAFVLGKAFQMSKIEALA	I	LICGCSPGGNMSNLF	108
Rabbit	ENSOCUP00000008010	61	LAIALVAQYGIMPLTAFVLGKVFRMNNIEALA	I	LVCGCSPGGNMSNLF	108
Brazilian guinea pig	ENSCAPP00000009872	61	LAIAMVAQYGIMPLIAFLLGKVFSLTNIEALA	I	LICGCSPGGNLSNIF	108
Chinese hamster CriGri	ENSCGRP00000012937	61	VVIAMLSQYGIMPLAAFVLGKAFHLKPIEALA	I	LICGCSPGGNLSNLF	108
Chinese hamster CHOK1GS	ENSCGRP00001018980	61	VVIAMLSQYGIMPLAAFVLGKAFHLKPIEALA	I	LICGCSPGGNLSNLF	108
Damara mole rat	ENSFDAP00000016479	68	ITIGFLCQFGIMPLTGFVLSVAFGILPVQAVV	V	IMGCCPGGASSNVV	115
Degu	ENSODEP00000018011	61	LTIAMVAQYGIMPLAAFLLGKAFRLSNIEALT	L	LICGCSPGGNLSNLF	108
Golden Hamster	ENSMAUP00000013002	61	VVIAMLSQYGIMPLAAFVLGKVFHLKPIEALA	I	LICGCSPGGNLSNLF	108
Guinea Pig	ENSCPOP00000005370	61	LAIAMVAQYGIMPLTAFLLGKVFSLTNIEALA	I	LICGCSPGGNLSNIF	108
Kangaroo rat	ENSDORP00000008282	69	IFVGFLCQFGIMPLMGFILSVAFHIIPIQAVV	v	LIMGCCPGGTASNVL	116
Long-tailed chinchilla	ENSCLAP00000006689	61	LAVAMVAQYGIMPLTAFALGKMFRLNNIEALA	I	LICGCSPGGNLSNIF	108
Naked mole-rat male	ENSHGLP00100024369	61	LATAMVAQYGIMPFSAFMLGKVFRLNNIEALA	I	LICGCSPGGNLSNAF	108
Naked mole-rat female	ENSHGLP00000013534	61	LATAMVAQYGIMPFSAFMLGKVFRLNNIEALA	I	LICGCSPGGNLSNAF	108
Northern American deer mouse	ENSPEMP00000022966	61	VIIAMLSQYGIMPLTAFVLGKVFRLNTIEALA	I	LICGCSPGGNLSNLF	108
Prairie vole	ENSMOCP00000016238	61	VIIAMLSQYGIMPLAAFVLGKVFRLNPIEALA	I	LICGCSPGGNLSNLF	108
Rat	ENSRNOP00000007825	61	VIVALVAQFGIMPLAAFLLGKIFHLSNIEALA	I	LICGCSPGGNLSNLF	108
Lesser Egyptian jerboa	ENSJJAP00000005005	61	LIIALVSQYGIMPLTAFVLGKVFRLTNIEALA	I	LICGCSPGGNLSNVF	108
Squirrel	ENSSTOP00000003472	61	LAIAMVAQYGIMPLTAFVLGKVFQLNNIEALA	I	LICGCSPGGNLSNIF	108
Upper Galilee mountains blind mole rat	ENSNGAP00000000640	61	LLIAMVAQYGIMPLAAFVLGKVFKLSNIEALA	I	LICGCSPGGNLSNLF	108

Other mammals	Peptide	From	Amino acid sequence			To
Cat	ENSFCAP00000008204	61	LAIALIAQYGIMPLTAFALGKVFQLNNIEALA	I	LVCGCSPGGTLSNIF	108
Dog	ENSCAFP00000024409	61	LVIALIAQYGIMPLTAFTLGKVFRLNNIEALA	I	LVCGCSPGGTLSNVF	108
Ferret	ENSMPUP00000007411	61	LAIALMAQYGIMPLTAFTLGKVFRLNNIEALA	I	LVCGCSPGGTLSNIF	108
Panda	ENSAMEP00000015380	61	LAIALIAQYGIMPLTAFTLGKVFRLNNIEALA	I	LVCGCSPGGTLSNVF	108
Alpaca	ENSVPAP00000005985	61	LAIALVAQYGIMPLTAFSLGKLFRLNNIEALA	I	LICGCSPGGNLSNVF	108
Cow	ENSBTAP00000002446	61	LAVALVAQFGIMPLTAFGLGKFFQLNNVEALA	I	LICGCSPGGNLSNVF	108
Dolphin	ENSTTRP00000005928	68	IAAGLLCQFGLMPLTAYLLVISFSLKPVQAMA	I	LIMGCCPGGTISNIF	115
Horse	ENSECAP00000012659	61	LAIALVAQYGIMPLTAFALGKVFQLNNVEALA	I	LVCGCSPGGNLSNIF	108
Pig	ENSSSCP00000002511	61	LAIALVAQYGIMPLTAFALGKLFRLNNVEALA	I	LICGCSPGGNLSNIF	108
Sheep	ENSOARP00000022773	61	LAVALVAQFGIMPLTAFGLGKFFQLNNVEALA	I	LICGCSPGGNLSNIF	108
Armadillo	ENSDNOP00000004782	61	LVIALVAQYGIMPLTAFVLGKVFRLNNIEALA	I	LICGCSPGGTLSNVF	108
Elephant	ENSLAFP00000017315	61	LVVALVAQYGLMPLTAFGLGKLFRLNNVEMLA	I	LICGCSPGGNLSNIF	108
Hedgehog	ENSEEUP00000005963	68	IFVGFLCQFGIMPLTGFILSVAFGILPLQAVV	V	LIMGCCPGGTASNIL	115
Hyrax	ENSPCAP00000004134	61	LAIALVAQYGIMPLTAFALGKLFRLNNIEKLA	V	LICGCSPGGNLSNIF	108
Lesser hedgehog tenrec	ENSETEP00000012656	61	LAIALVAQYGIMPLTAFSLGKLFRLNNIELLA	I	LICGCSPGGNLSNIF	108
Megabat	ENSPVAP00000009265	61	LAIALMARYGIMPLTAFVLGKVFRLNNIEALA	I	LICGSSPGGNLSNLF	108
Microbat	ENSMLUP00000010888	61	LAIAVVAQYGIMPLTAFALGKLFRLNNIEALA	I	LICGCSPGGNLSNVF	108
Shrew	ENSSARP00000000978	61	MIIAMVAQYGIMPLTAFSLGRIFKLNNIEALA	I	LVCGCSPGGNLSNIF	108
Sloth	ENSCHOP00000004848	61	LAIALVAQYGIMPLTAFALGKVFRLNNIEALA	I	LICGCSPGGNLSNVF	108
Tree Shrew	ENSTBEP00000002346	61	LAIALLAQYGIMPLTAFALGKVFPLNNIEALA	I	LVCGCSPGGNLSNVF	108
Opossum	ENSMODP00000014214	69	IIIALVSQYGVMPLTAFSLGKIFHLNNVEALA	I	LICGCSPGGTLSNIF	116
Platypus	ENSOANP00000017942	76	IIVGFLCQFGIMPLTGFILSLAFNVLPVQAVV	I	IIMGCCPGGTASNIL	123
Tasmanian devil	ENSSHAP00000003547	69	IAIALVSQYGVMPLTAFALGKIFQLNNIEALA	I	LICGCSPGGTLSNMF	116
Wallaby	ENSMEUP00000007961	61	IAIALVSQYGVMPLTAFALGKIFRLNNVEALA	I	LICGCSPGGTLSNIF	108

Other vertebrates	Peptide	From	Amino acid sequence	To
Anole lizard	ENSACAP00000005008	37	VMAAVSQFGIMMPLTAFALSKIFRLAPMEALT **V** FICGCCPGGTLSNIL	84
Chinese softshell turtle	ENSPSIP00000003539	76	ICVGFLCQFGIMPLTGFVLALLFNVLPAQAVA **V** LIMGCCPGGTSSNIL	123
Chicken	ENSGALP00000054869	70	IFVGFLCQFGIMPLTAFLLSLAFDVHPIQAVV **V** MIMGCCPGGTASNII	117
Duck	ENSAPLP00000014650	70	VAVAVMSQYGVMPLTAFVLGKLFQLGPTESLA **I** LICGCCPGGNLSNIF	117
Flycatcher	ENSFALP00000005168	62	VAVAIMAQYSIMPLTAFILGKLFQLGTSESLA **I** LICGCCPGGNLSSIF	109
Turkey	ENSMGAP00000012582	58	VLLALLGQFVAMPLVAFLLALIFALDEVAAVA **V** LLCGCCPGGNLSNLM	105
Zebra Finch	ENSTGUP00000011770	44	VAIAVLVQYSIMPLTAFILGKLFQLGTSESLA **I** LICGCCPGGNLSNIF	91
Amazon molly	ENSPFOP00000017822	79	VAIAILSQYGIMPLTAFCLTKGFQLSEATAVV **I** LICGCCPGGALSNIL	126
Cave fish	ENSAMXP00000012264	75	VAIAVVAQYGIMPLSAFSLAKLLQLGPMETVA **V** LVCGCSPGGNLSNIL	122
Cod	ENSGMOP00000017105	62	VAIAVVAQFGIMPLTAFCLATVFKLEQIEVLT **V** LICGCCPGGNLSNIF	109
Coelacanth	ENSLACP00000008119	76	MAIAVVAQYGIMPLTAFALAKAFKLPTIESVT **V** LICGCCPGGNLSNIF	123
Fugu	ENSTRUP00000037654	46	VAIALVAQFGIMPLTAFGLAKFLQMDAIKAVT **V** LICGCCPGGTLSNIF	93
Medaka	ENSORLP00000021052	101	VAIALLAQFFIMPLSAFCLAKVLQMDPIKAVT **V** LICGCCPGGTLSNIF	148
Platyfish	ENSXMAP00000007841	71	VAIAILSQYGIMPLTAFCLTKGFQVPEATAVV **I** LICGCCPGGAMSNIL	117
Spotted gar	ENSLOCP00000015305	76	VGIAVIAQYGVMPITAFALAKMFQLGAIEAVT **V** LICGCCPGGNLSNIF	123
Stickleback	ENSGACP00000010376	83	LIIAVVSQYVVMPLVAFCLVQAFQLTEIRAVV **V** LICGCCPGGNLSNIL	130
Tetraodon	ENSTNIP00000020983	72	IVVGFLCQFGIMPFTAFALALAFQVIPVQAVV **I** IIMGCCPGGTGSNII	119
Tilapia	ENSONIP00000007266	82	VAIAVVAQYGIMPLTAFCLAKLFKLAEIAAVV **I** LICGCCPGGALSNIL	129
Zebrafish	ENSDARP00000047825	72	VVIAVGAQYGIMPFTAFCLAKLFRLSPMESLS **V** LICGCCPGGNLSNIF	119
Xenopus	ENSXETP00000059800	69	IGIALFSQFGIMPLAAFSLAHVFQLNAIESVT **V** LICGCCPGGVLSSVF	116
Lamprey	ENSPMAP00000005222	76	IFIAVLAQFGIMPLTAFALAKVFRLNPVEAVT **V** LICGCCPGGNLSNVF	123

Other species	Peptide	From	Aminno acid sequence	To
Caenorhabditis elegans	Y71G10AR.4 pep	219	PGIGLFAQFIIMPLLSYLIAYAIFMPRGLYSM **A** LGLFVTGCSPGGGAS	266
Ciona intestinalis	ENSCINP00000008688	45	IAIAASCQFGIMPFISFLMAKIFGLDKVAAIA **V** LVTGCCPGGNLSNLL	93
Ciona savignyi	ENSCSAVP00000001569	61	IALVAQFLIMPASAFGLTQAFQLDTYAAIAVL **I** CGCCPGGNLSNMLAY	108
Fruitfly	FBpp0074072	188	PVVGVVSRFVLMPAVGFGLGRALWPDSWPLQLA **L** FYTALSPSGGLANVC	235

Supplementary Figure 1: Comparative alignment of the homologous peptides in rodents and lagomorphs, other mammals, other vertebrates as well as other species.
